# Inhibition of keratinocyte ferroptosis suppresses psoriatic inflammation

**DOI:** 10.1038/s41419-021-04284-5

**Published:** 2021-10-27

**Authors:** Yanhong Shou, Lu Yang, Yongsheng Yang, Jinhua Xu

**Affiliations:** 1 Department of Dermatology, Huashan Hospital, Fudan University, Shanghai, P. R. China; 2 Shanghai Institute of Dermatology, Shanghai, P. R. China

**Keywords:** Cell death, Diseases

## Abstract

Psoriasis is a common, chronic, and recurrent inflammatory disease. It is characterized by hyperproliferation and abnormal differentiation of keratinocytes. Keratinocyte death is also involved in many pathophysiological conditions and amplifies the inflammatory cascade. As a newly recognized form of cell death, ferroptosis is involved in several inflammatory diseases. In this study, we aimed to investigate a previously unrecognized role for ferroptosis in psoriasis. Ferroptosis is mediated by lipid peroxidation and iron overload. Compared with normal lesions, the mRNA expression of acyl-CoA synthetase long-chain family member 4 (*ACSL4*), prostaglandin-endoperoxide synthase 2 (*PTGS2*), and transferrin receptor (*TFRC*) were highly expressed in psoriatic lesions, with decreased levels of glutathione peroxidase 4 (*GPX4*), ferritin light chain (*FTL*), and ferritin heavy chain 1 (*FTH1*). The protein levels of ACSL4 and GPX4 were consistent with their mRNA levels. A similar tendency of ferroptosis was also observed in erastin-treated human primary keratinocytes and the Imiquimod (IMQ)-induced model of psoriasis. To investigate the correlation between inflammation and peroxidation, we analyzed single-cell RNA-sequencing data and identified 15 cell types. There was a high correlation between the activity of the lipid oxidation and the Th22/Th17 response in keratinocytes at a single-cell level. Moreover, ferrostatin-1 (Fer-1), a potent inhibitor of lipid peroxidation, suppressed ferroptosis-related changes in erastin-treated keratinocytes and alleviated psoriasiform dermatitis of IMQ-induced models. Additionally, Fer-1 blocked inflammatory responses in vitro and in vivo, reducing the production of cytokines including *TNF-α*, *IL-6*, *IL-1α*, *IL-1β*, *IL-17*, *IL-22*, and *IL-23*. This study revealed an expression pattern of ferroptosis in which specific molecules enhance inflammatory reactions in psoriasis.

## Introduction

Psoriasis is a common, chronic autoimmune skin disease. It is characterized by the modification of the epidermis as a result of hyperproliferation of keratinocytes, excessive infiltration of immune cells, and accumulation of inflammatory cytokines [1]. Clinically, the prevalence of psoriasis ranges from 2 to 3% globally [2]. However, the pathogenesis of the disease is not fully understood. Multiple factors including genetic and environmental factors promote disease progression of psoriasis [1, 3]. Abnormal interactions between infiltrating immune cells and activated keratinocytes contribute to psoriatic skin inflammation. Immune cells and the “IL-17 axis” are implicated in the pathogenic mechanisms of psoriasis [4]. Keratinocytes were previously recognized as initiators in the inflammatory process. They play an essential role in the amplification of the inflammatory cascade, through the secretion of chemokines and cytokines [5–7]. Intrinsic alterations of epidermal keratinocytes make individuals more susceptible to exogenous triggers, which fuels the inflammatory process.

Destruction of the epidermal barrier is associated with abnormal cell differentiation and shortened cellular turnover time, which leads to impairment and death of keratinocytes. However, the precise mechanism of programmed cell death in psoriasis is not fully understood [8, 9]. Psoriatic keratinocytes possess an enhanced ability to resist apoptosis [9], while they are more susceptible to necroptosis [8]. Several reports indicate that necroptosis triggers psoriatic inflammation in keratinocytes by releasing damage-associated molecular patterns (DAMPs) and activating inflammasomes [8, 10]. As a new type of programmed cell death, ferroptosis is morphologically, biologically, and genetically observed distinct from other types of cell death [11, 12]. Multiple molecules participate in the process of ferroptosis by regulating the lipid peroxidation state and cellular iron level [13]. Glutathione peroxidase 4 (GPX4) inhibits ferroptosis through lipid hydroperoxides clearance. The expression or activity of GPX4 is influenced by glutathione (GSH), and GSH synthesis depends on the availability of cysteine [14]. Glutamate/cysteine antiporter solute carrier family 7 member 11 (SLC7A11) can mediate cysteine uptake and thereby affect GSH production [15]. In addition, Acyl-CoA synthetase long-chain family member 4 (ACSL4) is a central enzyme of polyunsaturated fatty acid-containing phospholipids synthesis, which contributes to lipid peroxidation and subsequent ferroptosis [16]. Emerging evidence suggests that ferritin light chain (FTL), ferritin heavy chain 1 (FTH1), and transferrin receptor (TFRC) are involved in iron storage, iron entry, and iron homeostasis [17]. Abnormal increases in cellular Fe^2+^ contribute to Fenton-type reactions and uncontrolled lipid autoxidation. Additionally, prostaglandin-endoperoxide synthase 2 (PTGS2) is considered a typical potential biomarker for cells undergoing ferroptosis [18]. Due to its role in inflammation and tissue damage, ferroptosis has been reported in different diseases including nervous system diseases and heart diseases [19–22]. Mycobacterium tuberculosis infection induced the ferroptotic cell death, indicating a potential target to treat tuberculosis [23]. However, the role of ferroptosis in psoriasis has not been described. Ferroptosis is recognized as more immunogenic than apoptosis. This is because it not only promotes cell death but also potentiates a series of inflammatory reactions through the release of DAMPs and alarmins [24, 25]. Currently, ferroptosis inhibitors have been demonstrated to perform anti-inflammatory effects in experimental models of acute kidney injury, intracerebral hemorrhage, and neurodegenerative diseases [20, 26, 27]. Thus, the involvement of ferroptosis in the pathogenesis of psoriasis needs further investigation. The objective of this study was to investigate the underlying molecular mechanism of ferroptosis and its role in the pathogenic process of psoriasis; this provides a homeostatic mechanism to prevent aberrant cell death and inflammation.

## Results

### Ferroptosis-related cell death is activated in psoriasis lesions

**Fig. 1:**
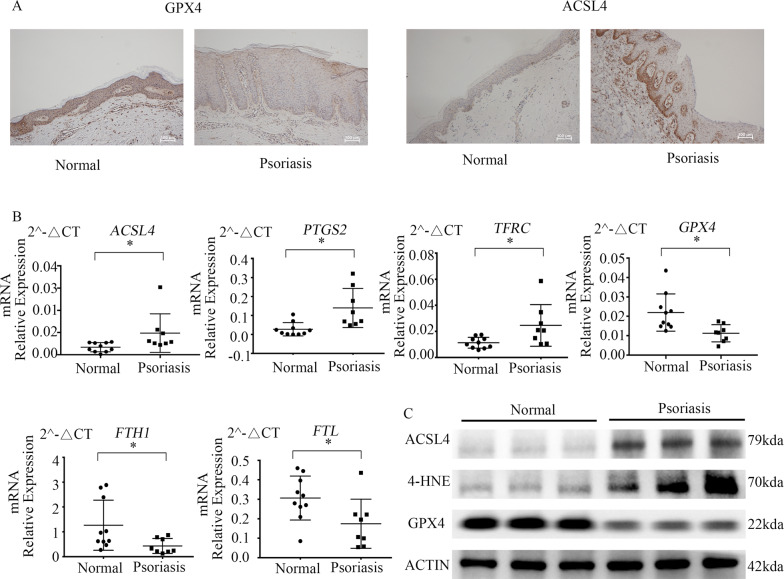
Ferroptosis is executed in psoriasis lesions. **A** Representative immunohistochemical staining for GPX4 and ACSL4 expression in normal lesions and psoriasis lesions. The white scale bar was 100 μm. **B** mRNA expression of *ACSL4*, *PTGS2*, *TFRC*, *GPX4*, *FTH1*, *and FTL* in normal samples (*n* = 10) and psoriatic lesions (*n* = 8). Data were normalized to actin mRNA expression. *P < 0.05. **C** Western blot for GPX4, 4-HNE-modified protein levels, and ACSL4 protein in normal lesions and psoriasis lesions. Actin was used as the loading control. ACSL4 acyl-CoA synthetase long-chain family member 4, GPX4 glutathione peroxidase 4, 4-HNE 4-hydroxynonenal, PTGS2 prostaglandin-endoperoxide synthase 2, TFRC transferrin receptor, FTL ferritin light chain, FTH1 ferritin heavy chain 1.

The expression of GPX4 and ACSL4 were detected in psoriasis lesions and normal samples by immunohistochemistry (Fig. 1A). In normal samples, GPX4 was significantly expressed in all layers of the epidermis, whereas in the psoriasis skin, GPX4 was barely expressed. ACSL4 was strongly expressed in the basal layer of the psoriatic epidermis relative to the normal skin. Quantitative RT-PCR studies showed a significant increase in the expression of *ACSL4*, *PTGS2*, and *TFRC*, and a decline of *GPX4*, *FTH1*, and *FTL* in psoriatic samples in comparison with normal samples (*P* = 0.039, *P* = 0.0212, *P* = 0.0214, *P* = 0.011, *P* = 0.040, *P* = 0.033, Fig. 1B). Western blot indicated the expression of ACSL4 and GPX4 in these skin tissues paralleled the appearance of the transcription expression levels. The by-product of lipid peroxidation, 4-hydroxynonenal (4-HNE), was also elevated in psoriatic lesions (Fig. 1C).

### Keratinocytes of psoriasis lesions exhibit lipid peroxidation

**Fig. 2:**
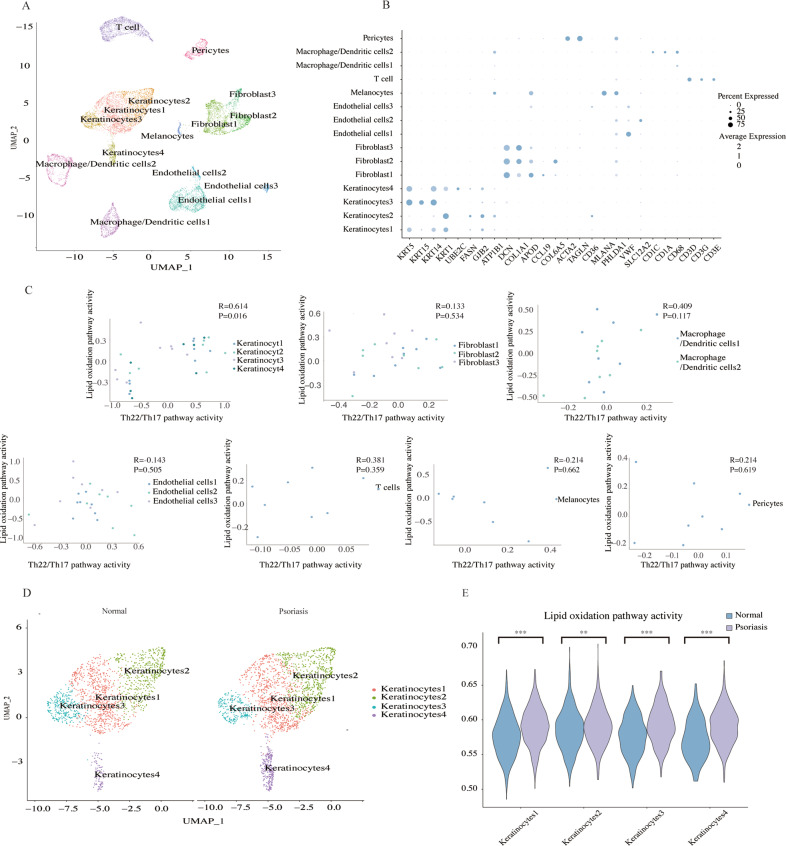
Lipid peroxidation in keratinocytes of psoriasis lesions is characterized at a single-cell level. **A** UMAP clustering of 12,025 cells by differentially expressed markers. **B** Gene expression patterns of marker genes for the cell subtypes. **C** The correlation between Th22Th17 pathway activity and lipid oxidation pathway activity in keratinocytes, fibroblasts, macrophage/dendritic cells, endothelial cells, melanocytes, and pericytes. **D** UMAP clustering of keratinocytes of normal and psoriatic samples. **E** Distributions of lipid oxidation pathway activity in different keratinocyte subtypes. **P < 0.01, ***P < 0.001. UMAP uniform manifold approximation and projection.

To further confirm the results of ferroptosis-related cell death in psoriatic lesions, single-cell RNA sequencing data (scRNA-seq) from eight samples were analyzed. After removing the batch effects, regressing out the effect of unique molecular identifiers (UMIs), and the quality filtering, 12,025 cells were filtered out and analyzed by graph-based clustering. Visualization of the 15 main clusters was depicted in a single UMAP plot (Fig. 2A), and markers of each cell type were shown in Fig. 2B.

As lipid peroxidation is essential for ferroptosis, we first sought to identify whether peroxidation is correlated with the inflammatory response in psoriasis. The activity of the lipid oxidation pathway and the Th22/Th17 pathway for each cell in the data was quantified using the AUCell package. We used the average level of each cluster as a surrogate of pathway activity level, and the correlation in each cell type was analyzed. Compared to fibroblasts, macrophage/dendritic cells, endothelial cells, T cells, melanocytes, and pericytes, the activity of lipid oxidation and the Th22/Th17 pathways were highly correlated in keratinocytes (Spearson’s *R* = 0.614, *P* = 0.016, Fig. 2C).

Next, we analyzed keratinocytes to investigate whether peroxidation is enhanced in human psoriatic samples when compared to controls (Fig. 2D). The lipid oxidation pathway was significantly upregulated in the four cell populations of keratinocytes from psoriasis samples (Fig. 2E). Taken together, these results revealed an enhancement of lipid peroxidation during psoriasis at the single-cell level.

### Keratinocytes are sensitive to ferroptosis

**Fig. 3:**
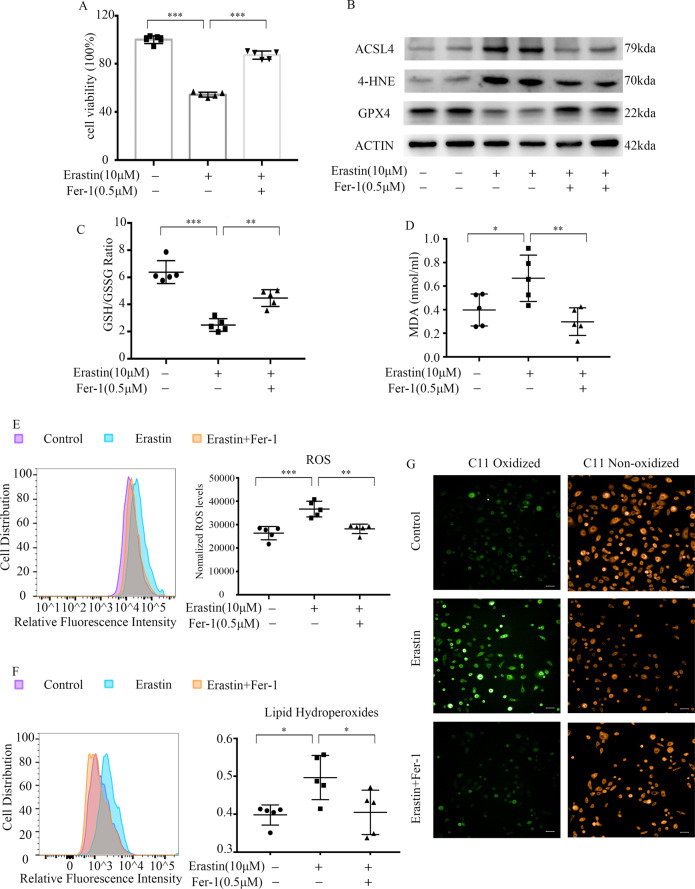
Keratinocytes are sensitive to ferroptosis. Keratinocytes were stimulated with 10 μM erastin in the absence or presence of the indicated inhibitor 0.5 μM Fer-1 for 24 h. **A** The CCK8 assay shows the viability of keratinocytes. **B** Western blot for GPX4, 4-HNE-modified protein levels, and ACSL4. Actin was used as the loading control. **C** GSH/GSSG changes seen in keratinocytes. **D** MDA level observed in keratinocytes. **E** Production of ROS observed by flow cytometry (left). Production of ROS was analyzed, and data were expressed as arbitrary units of DCF fluorescence (Right). **F** Generation of lipid hydroperoxides observed by flow cytometry (left). Levels of lipid hydroperoxides were expressed as BODIPY-C11Oxidized/ (BODIPY-C11Oxidized + BODIPY-C11Non-oxidized) ratio (Right). **G** Live-cell fluorescence imaging of lipid hydroperoxides in keratinocytes. Oxidized lipid is in green, a non-oxidized lipid is represented in orange. Scale bar, 20 μm. Values were presented as the mean ± standard error (*n* = 5). *P < 0.05,**P < 0.01,***P < 0.001. Independent experiments were repeated three times. ACSL4 acyl-CoA synthetase long-chain family member 4, GPX4 glutathione peroxidase 4, 4-HNE 4-hydroxynonenal, Fer-1 ferrostatin-1, MDA malondialdehyde, GSH glutathione, GSSG oxidized glutathione, ROS reactive oxygen species.

To assess whether keratinocytes are sensitive to ferroptosis signals, we treated them with the ferroptosis-inducing agent erastin. We then assessed their viability with the CCK8 assay. Our results show that the primary human keratinocytes (KCs) are sensitive to erastin-dependent ferroptosis stimulation in a time- and concentration-dependent manner (Fig. S1A–D). Furthermore, Fer-1, a specific inhibitor of ferroptosis, eliminated erastin-induced death (*P* < 0.001, Fig. 3A).

Figure 3B indicated that erastin could inhibit GPX4 protein expression, which may subsequently play a critical role in erastin-induced ferroptosis. ACSL4 and 4-HNE-modified protein levels were upregulated in erastin-treated keratinocytes and downregulated in Fer-1-pre-treated cells (Fig. 3B). As expected, erastin caused a dramatic decrease in GSH, leading to a decrease in the GSH/glutathione disulfide (GSSG) ratio (Fig. 3C).

In our study, we also estimated the lipid peroxidation levels in keratinocytes by detecting malondialdehyde (MDA) content, ROS generation, and lipid hydroperoxides level. MDA, an end-product of lipid hydroperoxides, was increased in erastin-treated KCs and decreased in Fer-1 pre-treated cells (Fig. 3D). DCF fluorescence was used to measure the intracellular ROS level. C11-BODIPY probe was employed to estimate the amount of lipid hydroperoxides in cell membranes. ROS and lipid hydroperoxides accumulated in erastin-treated KCs (Fig. 3E, F). Accordingly, we found an increase in oxidized lipid on the surface of erastin-treated cells compared to controls (Fig. 3G). Together, these findings demonstrate that keratinocytes are sensitive to ferroptosis.

### Induction of ferroptosis facilities inflammation

**Fig. 4:**
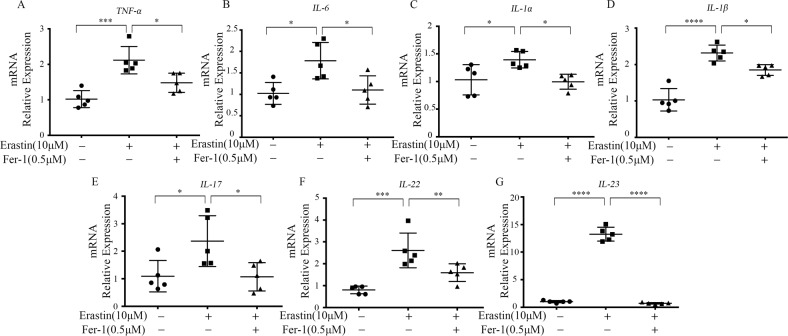
Inflammation levels changes in erastin-treated keratinocytes. **A**–**G** Keratinocytes were stimulated with 10 μM erastin for 24 h, with or without 0.5 μM Fer-1 treatment (*n* = 5). The mRNA expression of inflammatory cytokines including *TNF-α*, *IL-6*, *IL-1α*, *IL-1β*, *IL-6*, *IL-17*, *IL-22*, and *IL-23* was examined. Actin was used as the loading control. Values were presented as the mean ± standard error (*n* = 5). *P < 0.05, **P < 0.01, ***P < 0.001. Independent experiments were repeated three times. Fer-1 ferrostatin-1.

To confirm whether erastin-induced peroxidation and ferroptosis are related to the Th22/Th17 response, we examined keratinocytes for the change in inflammation levels in the presence of erastin or Fer-1. Quantitative RT-PCR was conducted to analyze the mRNA expression of inflammatory cytokines including *TNF-α*, *IL-6*, *IL-1α*, *IL-1β*, *IL-17*, *IL-22*, and *IL-23*. The expression of the inflammatory cytokines increased significantly after erastin stimulation and decreased after Fer-1 treatment (Fig. 4A–G).

### Fer-1 alleviates inflammation in mice with IMQ-induced psoriasiform dermatitis

**Fig. 5:**
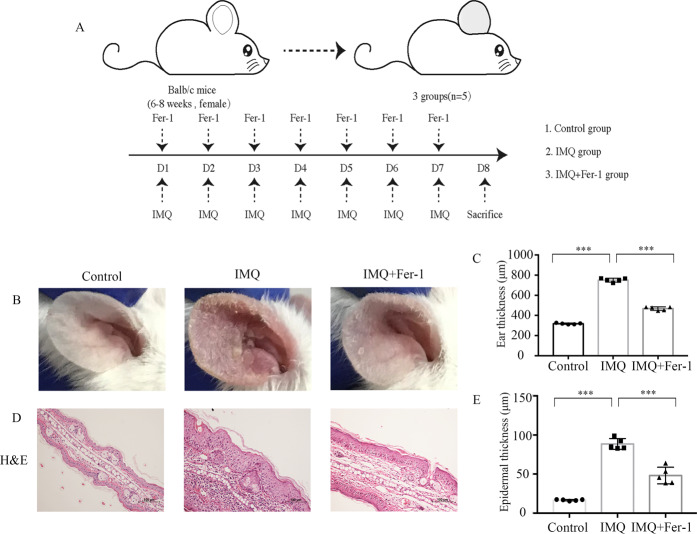
Fer-1 treatment attenuates IMQ-induced psoriasis-like dermatitis of mice. Mice were topical administered 5 mg IMQ cream or control cream on mouse ears for 7 consecutive days. An equal volume of Fer-1 (0.8 mg/kg/day) or 10% DMSO (50 μl) were painted on mouse ears 30 min before IMQ application. **A** Schematic representation of the animal experiments for the Control, IMQ, and IMQ + Fer-1 groups (*n* = 5). **B** Gross photograph of ears at day 8. **C** Ear thickness on day 8. **D** Hematoxylin and eosin staining of ear sections on day 8. Scale bar, 100 μm. **E** The thickness of the epidermis in ear sections was calculated using ImageJ software. Values were presented as the mean ± standard error (*n* = 5). ***p < 0.001. Independent experiments were repeated three times. IMQ imiquimod, Fer-1 ferrostatin-1.

To investigate whether ferroptosis signals contribute to psoriasis inflammation, a mouse model of psoriasis dermatitis was established via topical IMQ application. The ears of the mice were painted with either Fer-1 or DMSO 30 min before IMQ application every day (Fig. 5A). On day 8, skin inflammation signs were observed in the IMQ group, including erythema, increased skin thickness, and scales. Macroscopic observations indicated that pretreatment with Fer-1 significantly abated the phenotype of IMQ-induced psoriasis-like skin inflammation (Fig. 5B). We then took measurements of the thickness of the right ears of the mice. The ear thickness of the IMQ group increased significantly in comparison with the control group (318 vs. 749.2 mm, *P* < 0.001, Fig. 5C). Moreover, the ear thickness of the IMQ-Fer-1 group was significantly lower than that of the IMQ group (468 vs. 749.2 mm, *P* < 0.001, Fig. 5C). In the IMQ group, hematoxylin and eosin (HE) staining revealed obvious epidermal hyperplasia, dyskeratosis, and parakeratosis. Compared with the IMQ-treated mice, Fer-1 pretreatment significantly reduced the histologic change with the decrease of hyperplasia and dyskeratosis (Fig. 5D). The epidermal thickness of tissues stained with hematoxylin and eosin was calculated. The thickness was lower in the IMQ-Fer-1 group than that of the IMQ group (16.7 vs. 88.44 μm, *P* < 0.001, Fig. 5E). Therefore, Fer-1 reduced clinical manifestations in the IMQ-induced mouse model.

**Fig. 6:**
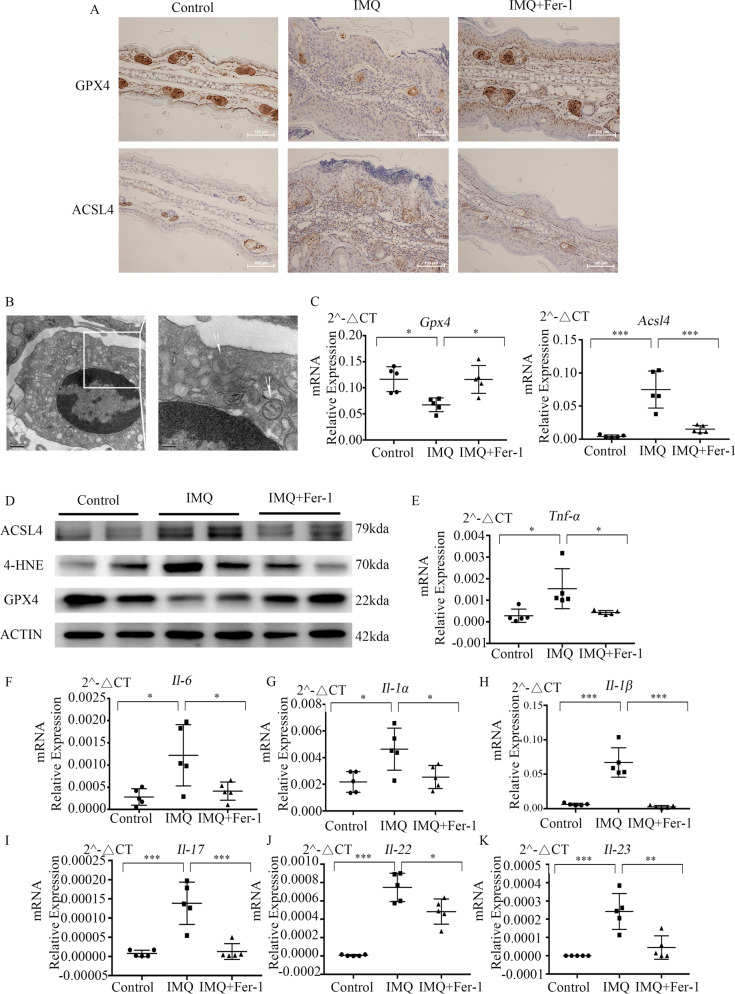
Fer-1 treatment inhibits ferroptosis and alleviates inflammation in mice with IMQ-induced dermatitis. **A** Representative immunohistochemical staining for GPX4 and ACSL4 expression in Control, IMQ, and IMQ + Fer-1 groups. The white scale bar was 100 μm. **B** Images of keratinocyte changes seen in the section from the IMQ group by electron microscopy. (Inset) Swollen mitochondria. White arrows indicate shrinkage of the mitochondria with dysmorphosis or vanishing of the mitochondrial cristae. Scale bar, 0.5 μm (Left), 200 μm (Right). **C** mRNA expression of *Gpx4* and *Acsl4* in Control, IMQ, and IMQ + Fer-1 groups. **D** Western blot result showing expression of GPX4 and ACSL4, and 4-HNE-modified protein levels in Control, IMQ, and IMQ + Fer-1 groups. **E**–**K** Levels of mRNA expression of *Tnf-α*, *Il-6*, *Il-1α*, *Il-1β*, *Il-17*, *Il-22*, and *Il-23* in the ears on day 8. Results were presented as the mean ± standard error (*n* = 5). *P < 0.05, **P < 0.01, ***P < 0.001. Independent experiments were repeated three times. ACSL4 acyl-CoA synthetase long-chain family member 4, GPX4 glutathione peroxidase 4, 4-HNE 4-hydroxynonenal, IMQ imiquimod, Fer-1 ferrostatin-1.

Immunohistochemical analysis showed that ACSL4 was strongly expressed in the basal epidermal layer in the IMQ group, while GPX4 was significantly reduced (Fig. 6A). In ear lesions of IMQ-treated mice, ferroptotic characteristic changes of mitochondria was observed in keratinocytes in the electron microscope. White arrows indicate shrinkage of the mitochondria with dysmorphosis or vanishing of the mitochondrial cristae (Fig. 6B). Moreover, Quantitative RT-PCR and western blot were used to examine the expression of key molecules in ferroptosis. We found that skin lesions of IMQ- treated mice displayed a gene expression pattern that mimicked downregulation of GPX4 and upregulation of ACSL4 and 4-HNE-modified protein levels in erastin-treated keratinocytes (Fig. 6C, D). However, the expression of GPX4 was restored, and the levels of ACSL4 and 4-HNE-modified protein levels were significantly suppressed by Fer-1.

To further verify the effects of Fer-1 on psoriasis inflammation in mice, we checked the expression of pro-inflammatory cytokines in the skin lesions of BALB/c mice. As shown in Fig. 6, *Tnf-α*, *Il-6*, *Il-1α*, *Il-1β*, *Il-17*, *Il-22*, and *Il-23* was significantly decreased in the IMQ-Fer-1 group compared to those of the IMQ group (Fig. 6E–K). So, Fer-1 exhibited an antipsoriasis-like inflammatory responses effect.

## Discussion

Ferroptosis is associated with multiple pathophysiological processes. Inhibition of ferroptosis has been considered as a novel target for some diseases [28]. The full picture of ferroptosis on the pathogenesis of psoriasis presumably is more complicated and further research is warranted. We here suggested that the induction of ferroptosis especially lipid peroxidation of psoriatic keratinocytes contributes to the inflammatory responses, which could be rescued by ferroptosis inhibitor Fer-1.

Previous studies implicated that psoriatic keratinocytes were resistant to apoptosis and produced inflammation-associated TNF-α [29]. Besides, necroptotic keratinocytes in psoriatic lesions had enhanced expression of receptor-interacting serine/threonine-protein kinase 1 and 3, and mixed lineage kinase domain-like protein, which regulated the distribution of S100, high mobility group box 1, IL-1β, and IL-18 cytokines. Necroptosis inhibitors such as necrostatin-1 and necrosulfonamide reduced inflammation and helped the clinical phenotypes in mouse models [8]. In addition, psoriasis was associated with abnormalities in lipid metabolism, free radical generation, and lymphokine production [30–32]. The levels of MDA, nitric oxide, ROS, and epidermal iron in psoriasis patients increased, while the levels of superoxide dismutase, glutathione peroxidase activity, GPX4 expression, and total antioxidant capacity decreased [33–37]. These levels were correlated with the severity of the disease. As mentioned previously, the paradigm of ferroptosis involves lipid peroxidation accumulation and iron overload. In our study, we found significant elevation of ACSL4 and 4-HNE-modified protein levels, and reduction of GPX4 in psoriatic lesions, which have been reported to be essential regulators under the process of ferroptosis [38]. Furthermore, elevated mRNA levels of *TFRC* and reduced levels of *FTH1* and *FTL* demonstrated increased intracellular labile iron to some extent. Additionally, *PTGS2* mRNA was elevated in the psoriatic lesions, which has been studied as a pharmacodynamic biomarker of ferroptotic conditions [18]. Thus, ferroptosis was putatively activated in psoriatic lesions.

Ferroptosis have been demonstrated to trigger and amplify the inflammatory responses [21, 39–41]. As a non-apoptotic form of programmed cell death, ferroptosis is associated with the release of DAMPs and alarmins [24]. Cyclooxygenase-2 (COX-2), encoded by the *PTGS2* gene, accelerates the metabolism of arachidonic acid and amplifies inflammation through the secretion of inflammatory signaling molecules [24]. Besides, 4-HNE, a major lipid peroxidation-derived aldehyde, can inhibit critical prosurvival molecules and thereby enhance ferroptosis [38]. It is largely responsible for the upregulated expression of COX-2. This indicates that 4-HNE links ferroptosis and chronic inflammation through the activation of cyclooxygenase. In addition, the release of IL-33 and other unidentified pathways are also found to function in ferroptosis-related inflammation [42]. Accordingly, we speculated that there is a complex link between ferroptosis and inflammation in psoriatic lesions.

In our research, a positive correlation was observed between lipid oxidation and the Th22/Th17 pathway at a single-cell level. To further elucidate the molecular mechanism, we assessed the changes in erastin-stimulated keratinocytes and IMQ- treated mice ear. There were upregulated lipid peroxidation-related markers and inflammatory cytokines in both cellular and ex vivo models. Currently, different antioxidants exhibited anti-inflammatory effects in experimental models and diseases, including neurodegeneration [43], acute renal failure [44], ischemia/reperfusion injury [45], Huntington’s disease, and periventricular leukomalacia [46]. Here, Fer-1 functioned successfully in the remission of inflammatory reactions, including skin thickness, scales, and cytokines production. It was therefore plausible that inflammatory responses were amplified by lipid peroxidation, which might provide a particular therapeutic target.

A “chicken or the egg” situation arises if we consider the initiation between ferroptosis and inflammation. Topical application of erastin or RSL3 in ears of mice did not exacerbate the characteristic features of IMQ-induced psoriasiform dermatitis (see Fig. S2). Additionally, ferroptotic characteristic changes of mitochondria were observed in IMQ- treated mice ear, suggesting ferroptosis occurrence in IMQ-induced psoriasiform dermatitis. It cannot be ignored that inhibition of ferroptosis with Fer-1 is beneficial for the treatment of IMQ-induced psoriasis-like dermatitis, especially from the first day onwards until the end of the study (see Fig. S3). This partly shed light on the role of ferroptosis in inflammation. Ferroptosis was presumably not an initiating factor to trigger psoriasis-like dermatitis but appeared in the disease and strengthen inflammation.

The reason why GPX4 expression decreased in psoriatic lesions was unclear. One possible explanation is the presence of depressed selenium status in patients with psoriasis [47]. Selenium depletion affects the biosynthesis of GPX4 and subsequent antioxidative activity [48]. In addition to selenium deficiency, elevated levels of the mechanistic target of rapamycin (mTOR) signaling proteins have been reported in psoriatic skin [49]. Several studies suggest mTOR complex 1 (mTORC1) inhibition sensitizes cells to ferroptosis by suppressing GPX4 synthesis, another recent study shows that mTORC1 inactivation attenuated ferroptosis [50, 51]. Here, *mTORC1* mRNA expression increased in psoriatic lesions, which was probably linked to GPX4 suppression (see Fig. S4). The transport of inorganic ions and amino acids pathway related to psoriasis risk includes the *SLC7A11* gene [52]. In our research, upregulated *SLC7A11* mRNA expression was in alignment with the previous study [35](see Fig. S4). Further studies are required to clarify these roles in regulating ferroptosis in psoriasis.

There remained shortages in this study. Certainly, the possibility that other cells in psoriatic lesion can exert ferroptosis cannot be ignored. In this study, our analysis at a single-cell level established the relationship between lipid oxidation and Th22/Th17 responses in keratinocytes and in other cell types. These correlations supported the importance of lipid peroxidation of keratinocytes to some degree. Secondly, this study did not compare the anti-inflammatory effects of Fer-1 with those of the other ferroptosis inhibitors in vivo. Additionally, Fer-1 is a quite potent lipid peroxidation inhibitor and produces an anti-ferroptotic effect. But it still remains unclear how Fer-1 rescues GPX4 protein synthesis and suppress ACSL4 expression. Our results might be the basis for the lipid peroxidation and inflammatory responses mimicking to some extent.

In conclusion, we describe a previously unexplored pathogenic role of ferroptosis in sensitizing Th22/Th17-type cytokines, thereby providing candidate markers of psoriasis aggravation. Notably, inhibition of ferroptosis with Fer-1 is beneficial for the treatment of psoriasis. Likewise, defining the critical pathogenetic mediators of the process might provide new and more specific therapeutic targets for various ferroptosis-related diseases.

## Methods

### Patients and samples

A total of 18 skin biopsy samples was included: Ten samples from control patients and eight samples from psoriasis patients. This study was approved by the local ethics committee and the institutional review board of Huashan hospital.

### Data collection

The dataset for GSE150672 [53] was downloaded from the Gene Expression Omnibus (GEO) database (http://www.ncbi.nlm.nih.gov/geo/). The count matrix for five patients with psoriasis and three healthy control subjects were used for further analysis.

### Analysis of scRNA-seq

Eight single-cell transcriptome profiles were integrated based on the IntegrateData function in “Seurat” package [54]. To recognize main cell clusters, Uniform Manifold Approximation and Projection (UMAP) [55] with a resolution of 0.4 was performed. The FindAllMarkers function in Seurat was executed to identify marker genes. Subsequently, the expression of known cell-type markers was used to identify keratinocytes.

### Pathway activity calculation

The hallmark gene set of lipid peroxidation pathway was obtained from the GO database (http://geneontology.org/page/go-database). The major genes in the Th22/Th17 pathway were summarized based on the published articles. Genes used in this pipeline were listed in Supplementary Table S1. The activity of individual cells for each gene set was calculated via the AUCell package (version 1.8.0) [56]. Data were processed in AUCell using the AUCell_buildRankings function. The resulting rankings, along with the gene lists, were then put into the function AUCell_calcAUC (aucMaxRank set to 100% of the number of input genes).

### Mice

Female BALB/c-Mice at 6–8 weeks of age were purchased from Shanghai Jiesijie Experimental Animal Co. Ltd. Imiquimod (IMQ) cream was obtained from Mingxin Pharmaceutical Company (Sichuan, China). BALB/c mice were randomized selected and placed into different groups (*n* = 5). Ferrostatin-1 (Fer-1, HY-100579, MCE, China) was dissolved in 10% dimethyl sulfoxide (DMSO, Sigma, USA) and 90% ethanol. An equal volume of Fer-1 (0.8 mg/kg/day) or 10% DMSO was painted on mouse ears 30 min before IMQ application. We conducted topical application of 5 mg IMQ cream (containing 5% IMQ) or control cream on mouse ears for 7 consecutive days was conducted [57]. On day 8, the thickness of the ears was measured with a micrometer. Skin tissues were taken from the sacrificed mice for extraction of RNA or protein, and immunohistochemistry.

### Histology and immunohistochemistry

Paraffin-embedded tissues were cut into 3-μm sections. Hematoxylin-eosin (HE) staining was performed for histological examination and evaluation of epidermal thickness. Ten fields of view were randomly selected for each mouse and means of epidermal thickness were calculated using ImageJ software. For immunochemistry, the following primary antibody was used: Anti-GPX4 (1:400, ab125066, Abcam, USA), Anti-ACSL4 (1:800, ab155282, Abcam, USA). Then it was incubated with the secondary antibody for 30 min at room temperature. After washing with PBS, staining with diaminobenzidine (Rabbit specific HRP/DAB Detection IHC Kit, ab64261, Abcam, USA) was performed for 5 min. This was followed by counterstaining with hematoxylin, dehydration, and observation under a microscope.

### Cell culture and analysis

Primary human epidermal keratinocytes were isolated from the foreskin and cultured in a keratinocyte-sera-free medium (C-20011, Promocell, Germany). The culture medium was supplemented with epidermal growth factor and bovine pituitary extract. Second- to third-passage keratinocytes were transferred into six-well plates with a starting cell number of 2 × 10^5^. The keratinocytes were treated with10 μM of erastin (HY-15763, MCE, China), and 0.5 μM of Fer-1 to inhibit ferroptosis. The cells were incubated with 10 μl/well cell Counting Kit-8 (DOJINDO, CK04-13, Japan) solutions for 4 h at 37 °C. The optical density was examined at 450 nm using a microplate reader.

### Analysis of GSH/GSSG ratio

Keratinocytes were seeded into a six-well plate at 2 × 10^5^ cells per well. After the applied treatment, cells were lysed. GSH and GSSG were measured using the GSSG/GSH Quantification Kit (G263, Dojindo, Japan). The ratio of GSH to GSSG was determined based on the formula [ratio = (GSH − 2GSSG)/GSSG].

### Measurement of ROS generation

Production of reactive oxygen species (ROS) was detected using the Reactive Oxygen Species Assay Kit (S0033, Beyotime, China). The data were expressed as arbitrary units (AU) of DCF fluorescence measured by flow cytometry.

### Detection of malondialdehyde (MDA) content

According to the reaction with thiobarbituric acid, MDA was used as an index of lipid peroxidation. The content was measured using the Lipid Peroxidation (MDA) assay kit (A003-1, Jiancheng, China).

### Estimation of lipid peroxidation

Cells were incubated with the fluorescent probe C11-BODIPY (10 μm, D3861, Invitrogen, USA) for 30 min according to the manufacturer’s protocols. The reduced dye and the oxidized dye were detected at excitation/emission of 581/591 nm (Texas Red^®^ filter set) and 488/510 nm (traditional FITC filter set), respectively. Flow cytometry was performed to calculate the ratio of the emission fluorescence intensities at 590 to 510 nm. Confocal microscopy was conducted for observation of intracellular lipid peroxidation. Scale bar, 20 μm.

### Isolation of RNA from skin biopsy and quantitative real-time RT-PCR

RNA extraction of tissue samples and cells was performed using RNAiso Plus (TaKaRa, Japan). The extracted RNA (1 μg) was reverse transcribed to cDNA using PrimeScript^™^ RT Master Mix (Takara, Japan). Quantitative real-time polymerase chain reaction (PCR) based on SYBR Green fluorescence (TaKaRa, Japan) was performed using the 7500 Real-Time PCR System (Thermo Fisher Scientific, USA). The sequences of primers were listed in Supplementary Table S2. Data were analyzed by the delta Ct and delta-delta Ct method.

### Western blot analysis

Tissues and Cells were lysed in the lysis buffer (RIPA, Beyotime, China). The protein concentrations were measured by the BCA assay kit (Beyotime, China). Equal amounts of protein were loaded in 10% sodium dodecyl sulfate-polyacrylamide gel (SDS-PAGE) and were then transferred from the gel to polyvinylidene fluoride membranes (Millipore, USA). After blocking in a solution of 5% bovine serum albumin (BSA, Solarbio, China) for 1 h, the membranes were washed with TBST and then incubated with primary antibodies overnight at 4 °C. The following antibodies were used: anti-GPX4(1:2000), anti-4 Hydroxynonenal(1:1000, ab46545, Abcam, USA), anti-ACSL4(1:2000), anti-actin(1:2000, 4967, Cell Signaling Technology, USA). After washing, the membranes were incubated with horseradish peroxidase (HRP)-conjugated secondary antibodies for 1 h at 37 °C: goat anti-rabbit IgG antibody (1:2000, #7074, Cell Signaling Technology, USA). Bound antibodies were detected using the ECL western blotting detection system (Millipore, USA).

### Electron microscopy

Electron microscopy was used for the examination of the skin tissue. Skin sections were fixed by the addition of glutaraldehyde. The morphological changes were then observed using electron microscopy.

### Statistical analysis

All data were expressed as mean ± standard error, and significance was estimated by using one-way analysis of variance (ANOVA) tests, followed by the Tukey test. In some cases, unpaired *t*-tests were used. *P* values <0.05 were considered significant.

## Supplementary information

Supplementary Figure Legends

Supplementary Figure1

Supplementary Figure2

Supplementary Figure3

Supplementary Figure4

Supplementary Table

## Data Availability

The datasets analyzed during the current study are available through the accession number GSE150672 and the corresponding author on request.
