# Supplementary material for: Inhibition of keratinocyte ferroptosis suppresses psoriatic inflammation
Source: Cell Death Dis. 2021 Oct 27;12(11):1009. doi: 10.1038/s41419-021-04284-5 (PMC8551323; doi:10.1038/s41419-021-04284-5)
Supplement: Supplementary file 1 — Supplementary Figure Legends [file 41419_2021_4284_MOESM1_ESM.docx]

**Supplementary Figure 1**

(A and B) Erastin-induced concentration-dependent and time-dependent cell death in the CCK8 assay. (C and D) Keratinocytes were stimulated with different concentrations of erastin in 3h, 6h, 24h, 48h, 72h. Western blot for GPX4, 4-HNE, and ACSL4. Actin was used as the loading control. Values were presented as the mean±standard error (n=5). *P<0.05, **P<0.01, ***p<0.001 vs.control. Control represented untreated cells.

**Supplementary Figure 2**

Mice were topical administered 5 mg IMQ cream or control cream on mouse ears for 7 consecutive days. An equal volume of RSL3 or Erastin (0.8mg/kg/day) or 10% DMSO (50μl) were painted on mouse ears 30 minutes before IMQ application. (A) Schematic representation of the animal experiments for the Control, RSL3, Erastin, IMQ, IMQ +RSL3, and IMQ + Erastin groups (n = 5). (B) Gross photograph of ears at day 8. (C) Hematoxylin and eosin staining of ear sections on day 8. Scale bar, 100 μm. (D) Ear thickness on day 8.

(E) The thickness of the epidermis in ear sections was calculated using ImageJ software. Values were presented as the mean±standard error (n=5). ***p<0.001. *ns*, Not significant.

**Supplementary Figure 3**

Mice were topical administered 5 mg IMQ cream or control cream on mouse ears for 12 consecutive days. An equal volume of Fer-1 (0.8mg/kg/day) or 10% DMSO (50μl) were painted on mouse ears 30 minutes before IMQ application from 1st day, 5th day, and 9th day to the end of the study. (A) Schematic representation of the animal experiments for the Control, IMQ, IMQ+Fer-1(Day1), IMQ+Fer-1(Day5), and IMQ+Fer-1(Day9) groups (n = 5). (B) Gross photograph of ears at day 13. (C) Ear thickness on day 13. (D) Hematoxylin and eosin staining of ear sections on day 13. Scale bar, 100 μm. (E) The thickness of the epidermis in ear sections was calculated using ImageJ software. (F-K) Levels of mRNA expression of *Tnf-α*, *Il-1α*, *Il-1β*, *Il-6*, *Il-17*, *Il-22*, and *IL-23* in the ears on day 13. Values were presented as the mean±standard error (n=5). ***p<0.001. ns, Not significant.

**Supplementary Figure 4**

(A and B) mRNA expression of *mTORC1* and *SLC7A11* in normal samples (n=10) and psoriatic lesions (n=8). Data were normalized to actin mRNA expression. *P<0.05. (C and D) Keratinocytes were stimulated with 10μM erastin in the absence or presence of the indicated inhibitor 0.5μM Fer-1 for 24 h. mRNA expression of *GPX4* and *ACSL4* were detected. Actin was used as the loading control.
