# Supplementary figures and images for: Inhibition of keratinocyte ferroptosis suppresses psoriatic inflammation
Source: Cell Death Dis. 2021 Oct 27;12(11):1009. doi: 10.1038/s41419-021-04284-5 (PMC8551323; doi:10.1038/s41419-021-04284-5)

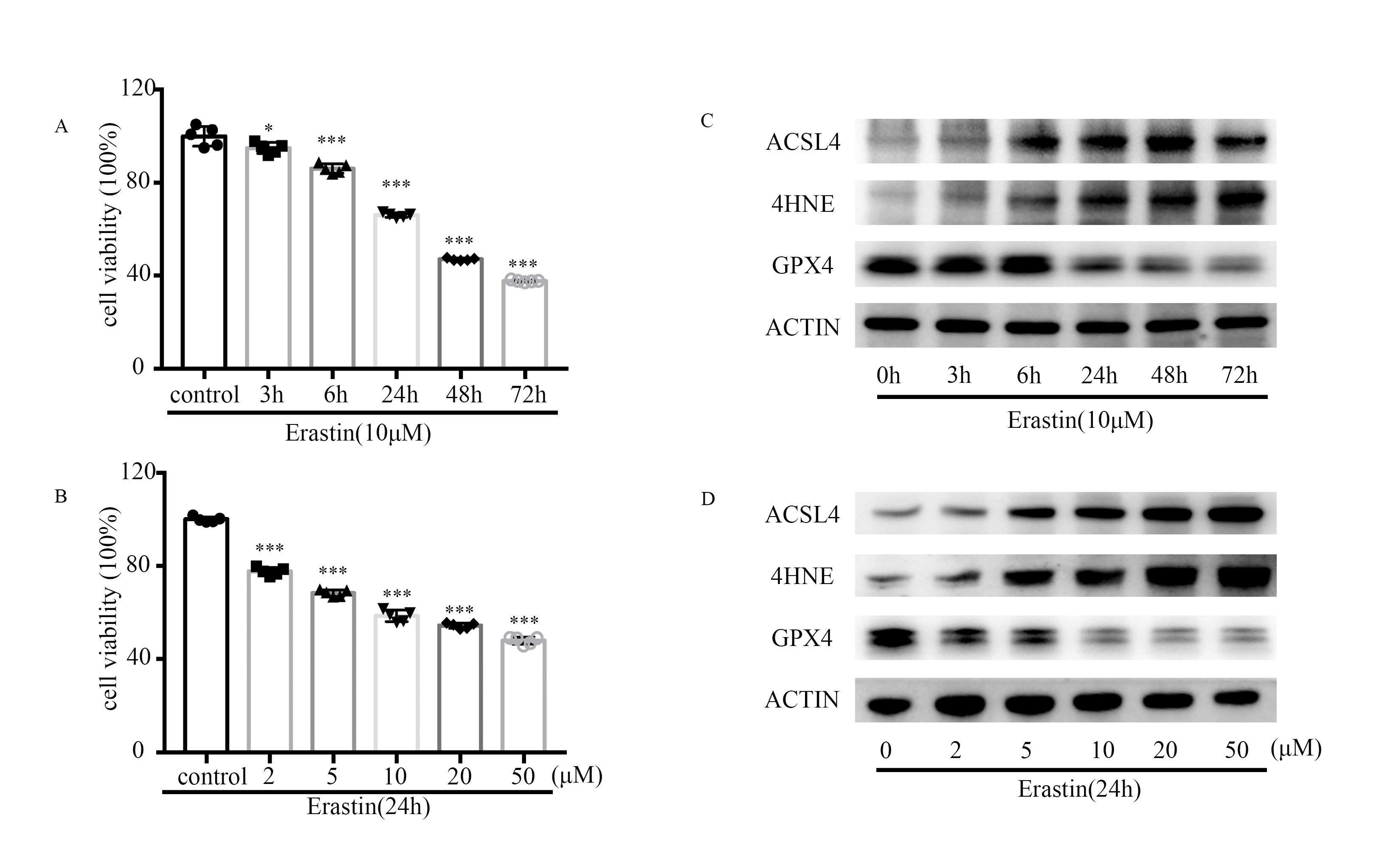

Supplement: Supplementary file 2 — Supplementary Figure1 [file 41419_2021_4284_MOESM2_ESM.tif]

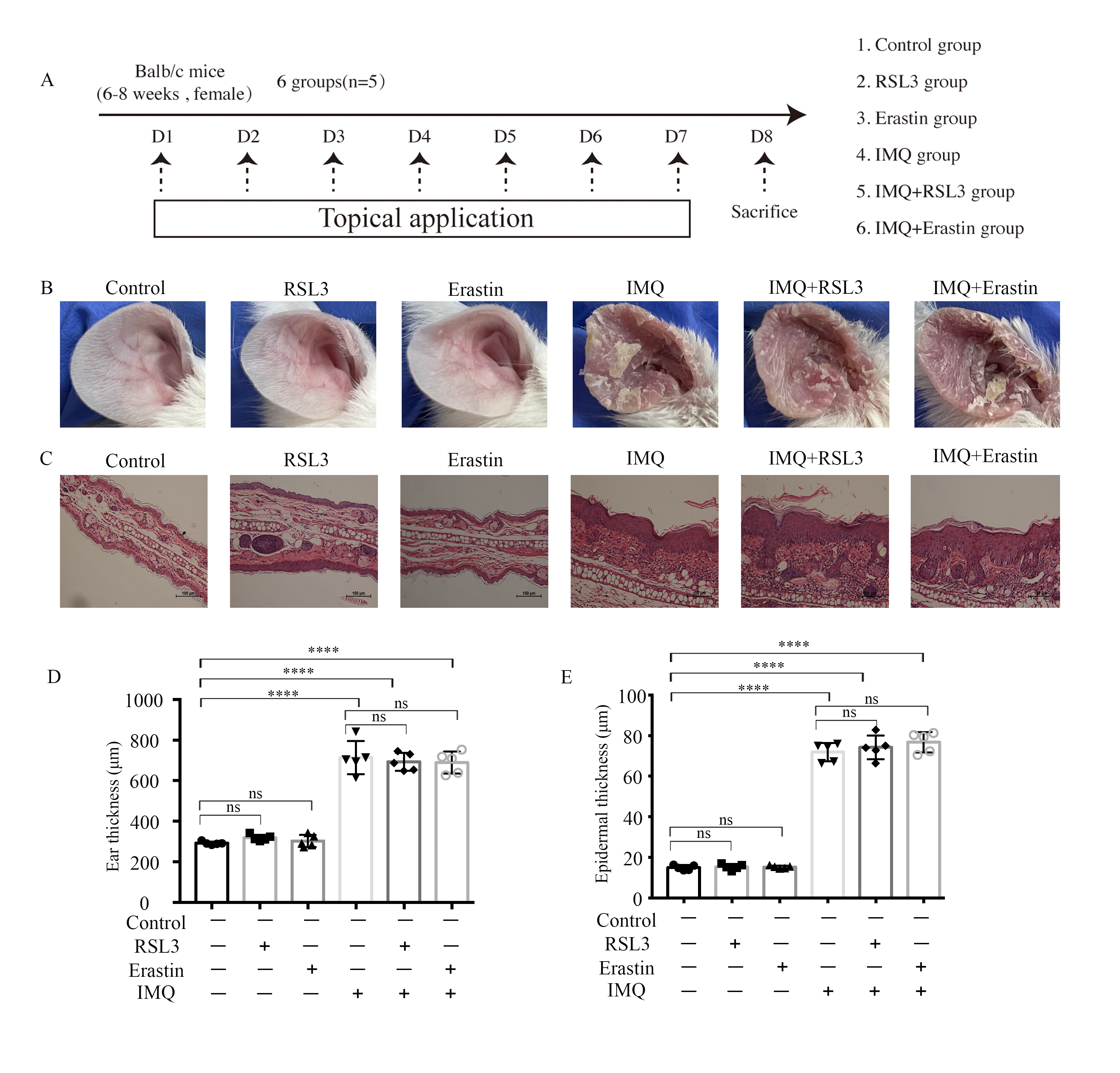

Supplement: Supplementary file 3 — Supplementary Figure2 [file 41419_2021_4284_MOESM3_ESM.tif]

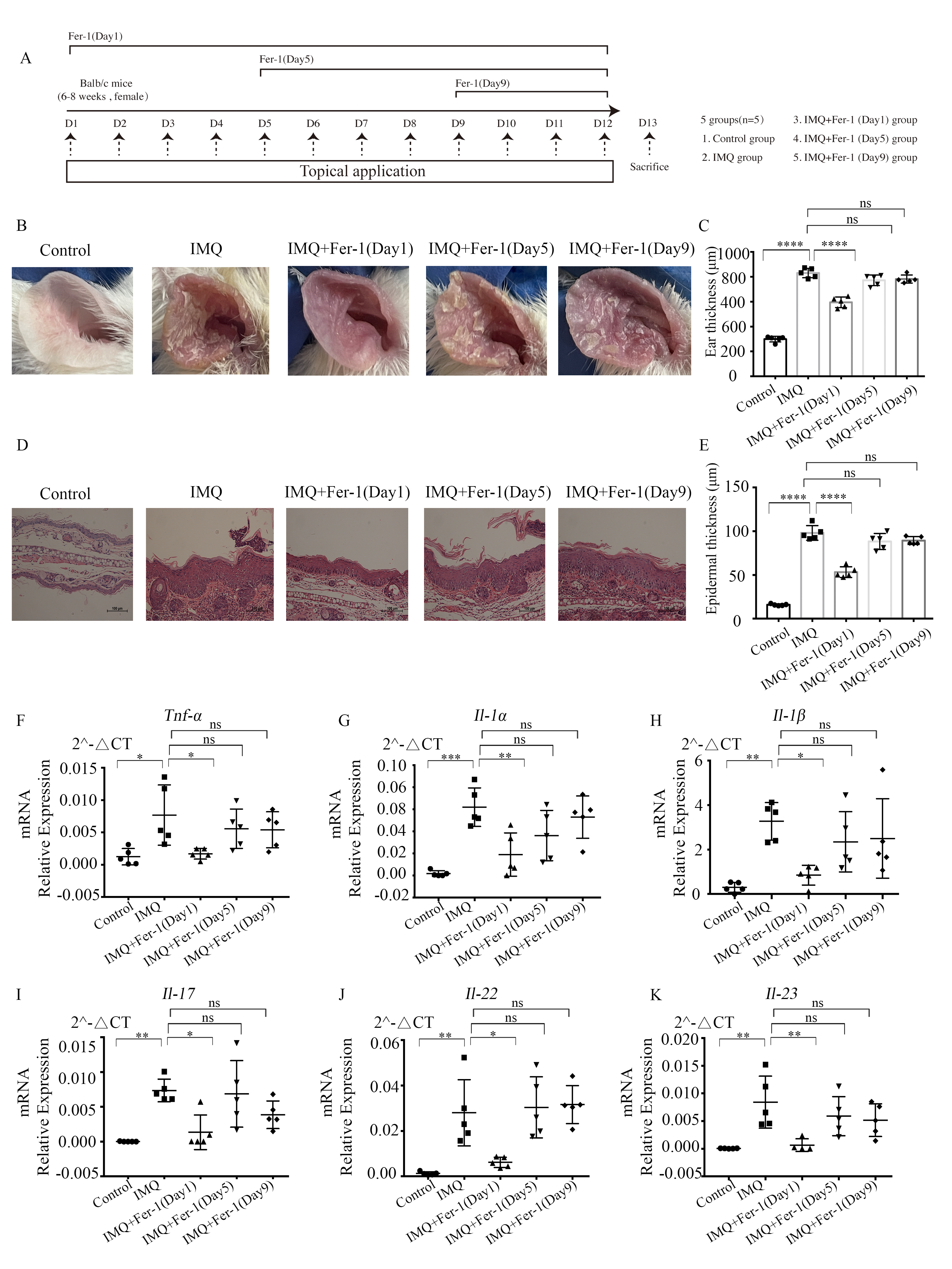

Supplement: Supplementary file 4 — Supplementary Figure3 [file 41419_2021_4284_MOESM4_ESM.tif]

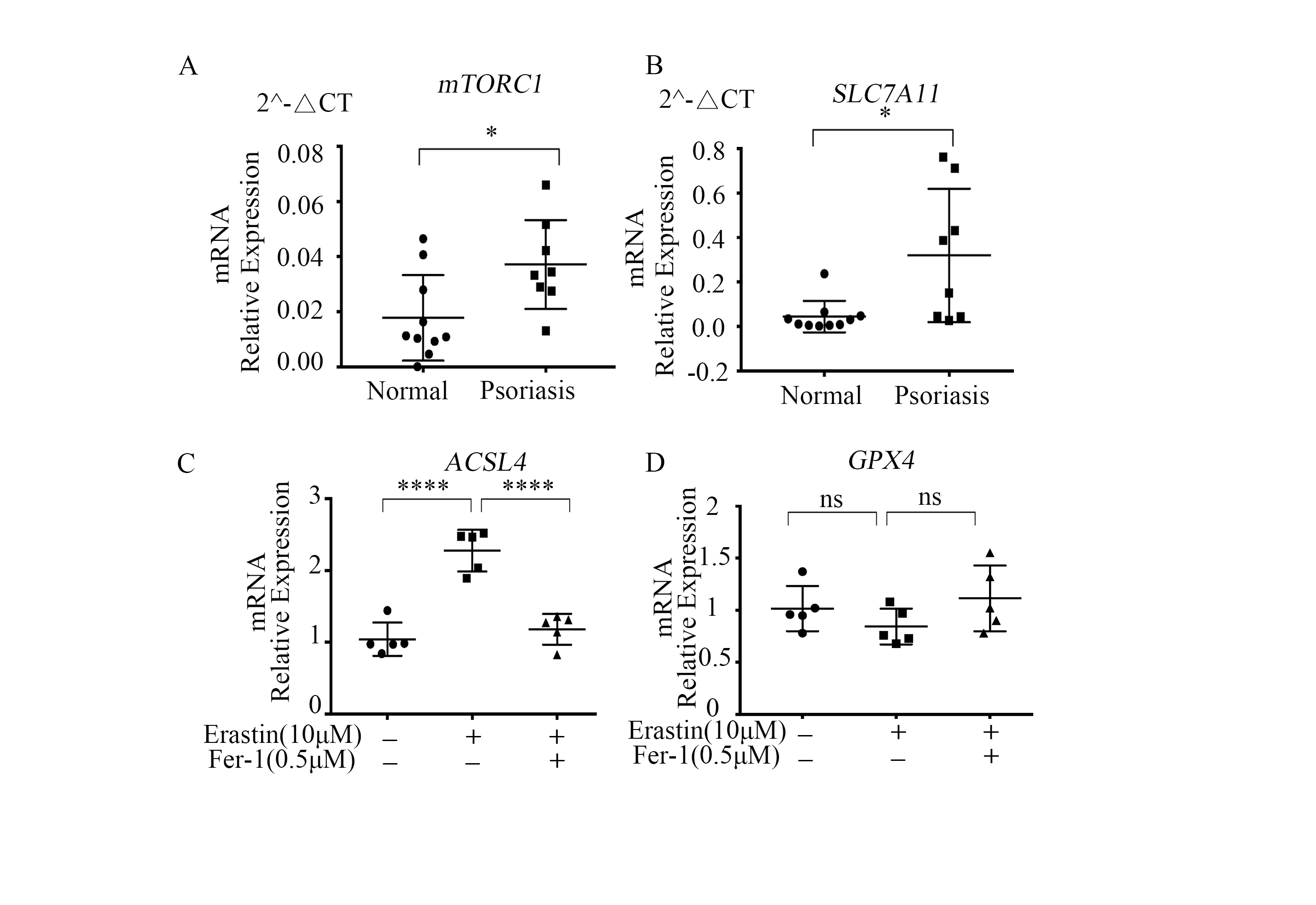

Supplement: Supplementary file 5 — Supplementary Figure4 [file 41419_2021_4284_MOESM5_ESM.tif]
