# Supplementary material for: Inhibition of keratinocyte ferroptosis suppresses psoriatic inflammation
Source: Cell Death Dis. 2021 Oct 27;12(11):1009. doi: 10.1038/s41419-021-04284-5 (PMC8551323; doi:10.1038/s41419-021-04284-5)
Supplement: Supplementary file 6 — Supplementary Table [file 41419_2021_4284_MOESM6_ESM.docx]

Supplementary TableS1 Genes used in Th22/Th17 pathway

| Th22Th17 | IL22 |
| --- | --- |
| Th22Th17 | S100A7 |
| Th22Th17 | S100A8 |
| Th22Th17 | S100A9 |
| Th22Th17 | S100A12 |
| Th22Th17 | IL17A |
| Th22Th17 | IL36G |
| Th22Th17 | IL23p19 |
| Th22Th17 | CCL20 |
| Th22Th17 | VNN3 |
| Th22Th17 | DEFB4A |
| Th22Th17 | LCN2 |

Supplementary Table S2 Primers used for quantitative reverse transcription polymerase chain reaction

| Primers(human) | Sequence (5′-3′) |
| --- | --- |
| Actin-F | TCATGAAGTGTGACGTGGACATC |
| Actin-R | CAGGAGGAGCAATGATCTTGATCT |
| TNF-α-F | AGCTGGTGGTGCCATCAGAGG |
| TNF-α-R | TGGTAGGAGACGGCGATGCG |
| IL-6-F | ACTCACCTCTTCAGAACGAATTG |
| IL-6-R | CCATCTTTGGAAGGTTCAGGTTG |
| IL-1α-F | TGGTAGTAGCAACCAACGGGA |
| IL-1α-R | ACTTTGATTGAGGGCGTCATTC |
| IL-1β-F | ATGATGGCTTATTACAGTGGCAA |
| IL-1β-R | GTCGGAGATTCGTAGCTGGA |
| IL-17-F | TCCCACGAAATCCAGGATGC |
| IL-17-R | GGATGTTCAGGTTGACCATCAC |
| IL-22-F | GCTTGACAAGTCCAACTTCCA |
| IL-22-R | GCTCACTCATACTGACTCCGT |
| IL-23-F | CTCAGGGACAACAGTCAGTTC |
| IL-23-R | ACAGGGCTATCAGGGAGCA |
| GPX4-F | GAGGCAAGACCGAAGTAAACTAC |
| GPX4-R | CCGAACTGGTTACACGGGAA |
| ACSL4-F | CATCCCTGGAGCAGATACTCT |
| ACSL4-R | TCACTTAGGATTTCCCTGGTCC |
| TFRC-F | ACCATTGTCATATACCCGGTTCA |
| TFRC-R | CAATAGCCCAAGTAGCCAATCAT |
| FTH1-F | CCCCCATTTGTGTGACTTCAT |
| FTH1-R | GCCCGAGGCTTAGCTTTCATT |
| FTL-F | CAGCCTGGTCAATTTGTACCT |
| FTL-R | GCCAATTCGCGGAAGAAGTG |
| PTGS2-F | CTGGCGCTCAGCCATACAG |
| PTGS2-R | CGCACTTATACTGGTCAAATCCC |
| mTORC1-F | ATGCTTGGAACCGGACCTG |
| mTORC1-R | TCTTGACTCATCTCTCGGAGTT |
| Primers(mouse) | Sequence (5′-3′) |
| Actin-F | GGAGATTACTGCCCTGGCTCCTA |
| Actin-R | GACTCATCGTACTCCTGCTTGCTG |
| TNF-α-F | CAGGCGGTGCCTATGTCTC |
| TNF-α-R | CGATCACCCCGAAGTTCAGTAG |
| IL-6-F | CTGCAAGAGACTTCCATCCAG |
| IL-6-R | AGTGGTATAGACAGGTCTGTTGG |
| IL-1α-F | TCTATGATGCAAGCTATGGCTCA |
| IL-1α-R | CGGCTCTCCTTGAAGGTGA |
| IL-1β-F | GAAATGCCACCTTTTGACAGTG |
| IL-1β-R | TGGATGCTCTCATCAGGACAG |
| IL-17-F | TCAGCGTGTCCAAACACTGAG |
| IL-17-R | CGCCAAGGGAGTTAAAGACTT |
| IL-22-F | ATGAGTTTTTCCCTTATGGGGAC |
| IL-22-R | GCTGGAAGTTGGACACCTCAA |
| IL-23-F | CAGCAGCTCTCTCGGAATCTC |
| IL-23-R | TGGATACGGGGCACATTATTTTT |
| GPX4-F | GCAACCAGTTTGGGAGGCAGGAG |
| GPX4-R | CCTCCATGGGACCATAGCGCTTC |
| ACSL4-F | CCTGAGGGGCTTGAAATTCAC |
| ACSL4-R | GTTGGTCTACTTGGAGGAACG |
